# The Impact of Storage Temperature and Time on Ergot Alkaloid Concentrations

**DOI:** 10.3390/toxins15080497

**Published:** 2023-08-05

**Authors:** Jensen E. Cherewyk, Taylor J. Grusie-Ogilvie, Sarah E. Parker, Barry R. Blakley, Ahmad N. Al-Dissi

**Affiliations:** 1 Department of Veterinary Biomedical Sciences, Western College of Veterinary Medicine, University of Saskatchewan, Saskatoon, SK S7N 5B4, Canada; brb237@mail.usask.ca; 2 Prairie Diagnostic Services (PDS), Saskatoon, SK S7N 5B4, Canada; taylor.ogilvie@pds.usask.ca; 3 Centre for Applied Epidemiology, Large Animal Clinical Sciences, Western College of Veterinary Medicine, University of Saskatchewan, Saskatoon, SK S7N 5B4, Canada; sarah.parker@usask.ca; 4 Department of Veterinary Pathology, Western College of Veterinary Medicine, University of Saskatchewan, Saskatoon, SK S7N 5B4, Canada; ahmad.aldissi@usask.ca

**Keywords:** contamination, grain, epimer

## Abstract

Ergot sclerotia produce toxic secondary metabolites, ergot alkaloids, that infect cereal crops and grasses. Ergot alkaloids have two isomeric configurations: the C-8-*R*-isomer (*R*-epimer), and the C-8-*S*-isomer (*S*-epimer). Ergot contaminated matrices, such as cereal grains or grasses, may be stored for extended periods at various temperatures before being analyzed, utilized, or consumed. This study assessed the concentration of six common ergot alkaloids in both configurations found in naturally contaminated wheat over time (one, two, and four months) at different temperatures (room temperature, +4 °C, and −20 °C) using ultra-high-performance liquid chromatography–tandem mass spectrometry. The data indicate that the total ergot concentration within a natural contaminated sample varies over time at room temperature, +4 °C, and −20 °C. The total ergot concentration increased until month two, and decreased at month four, independent of temperature (*p* < 0.05). The total *R*-epimer concentration appeared to be less stable over time than the total *S*-epimer concentration. The changes in the total *R* and total *S*-epimer concentrations may have been caused by changes in the ergocristine and ergocristinine concentrations, respectively. Time and temperature should be considered when storing potentially contaminated matrices in a laboratory or practical agriculture situations. Quantification of ergot contaminated matrices should occur prior to their use to ensure the most reliable estimates of the concentration of ergot.

## 1. Introduction

Ergot sclerotia contaminate cereal crops or grasses. If the infected crops or forage are harvested, food or feed quality may be impacted. Within recent years, samples contaminated with ergot sclerotia have increased in western Canada [1,2]. This increase in the incidence of ergot-contaminated samples may be associated with changing climate [3], or agricultural practices [1].

Ergot sclerotia contain toxic secondary metabolites known as ergot alkaloids [1]. Ergot alkaloids are divided into three structural classes: clavines, lysergic acid amides, and ergopeptines [4]. Each class exists in two configurations, the C-8-*R*-isomer and the C-8-*S*-isomer. Each configuration is known as the *R*-epimer and *S*-epimer, respectively, with either a -ine (*R*), or -inine (*S*) suffix. There are six common ergot alkaloids worldwide [5,6]. The six common *R* and six common *S*-epimers are, ergocornine/ergocorninine, ergocristine/ergocristinine, ergocryptine/ergocryptinine, ergometrine/ergometrinine, ergosine/ergosinine, and ergotamine/ergotaminine.

Ergot infected grains or grasses can be stored for months before they are used for food or feed products. The stability of ergot alkaloids in natural ergot contaminated samples has been studied [1,7,8,9,10]. The concentration of an individual ergot alkaloid, ergovaline, in infected tall fescue has been assessed over time at multiple temperatures [7,8,9,10]. Temperature and time appeared to decrease the concentration of ergovaline. In contrast, total ergot alkaloid concentration in rye was reported to be stable over days [11]. However, several studies demonstrated that total ergot alkaloid concentration was affected by longer time periods of months and years [1,8,10,12]. Study variability may be associated with differences in the time/temperature investigated. Discrepancies in the ergot alkaloids or epimers quantified may also contribute to the variation. Previous studies have not assessed the effects of time and temperature on the *R* and *S*-epimers separately.

The *R* and *S*-epimers of ergot alkaloids have demonstrated varied stability in solvents and raw material with altered experimental factors [13,14,15,16]. The *R* and *S*-epimers of ergot alkaloids can epimerize from one configuration to another [4]. Epimerization and degradation of ergot alkaloids can occur at high temperatures, greater than room temperature [13,16]. The effects of temperature lower than room temperature have been studied to a lesser extent. Certain *R* and *S*-epimers of ergot alkaloids are found at high concentrations worldwide [3,5,17,18,19]. All common ergot alkaloids should be analyzed in contaminated samples due to the potential concentration differences.

The concentration of ergot alkaloids in food and feed are determined for the health and safety of humans and animals. Safety standards on ergot alkaloids for human food [18,20] and animal feed [21] consumption have been established. The standards for ergot alkaloid concentration include only the *R*-epimer of ergot alkaloids and do not include the *S*-epimer in Canada at the present time. The European Union (EU) Commission Recommendation for ergot contaminated foodstuff includes both the *R* and *S*-epimers of ergot alkaloids in the regulatory standard [20]. The quantification of both *R* and *S*-epimers, under various storage conditions, should be conducted to prevent underestimation of the total ergot alkaloid concentration and provide an accurate estimate of exposure [4,17,18]. Recent studies have demonstrated that *S*-epimers may be bioactive and have toxic effects [22,23,24], further supporting the need for quantification of each configuration.

Samples suspected to be contaminated with ergot can be sent to laboratories to assess the concentration of ergot alkaloids [25]. Time and temperature may affect the concentration of ergot alkaloids in the submitted samples [7]. Various temperature conditions have been reported during sample storage after harvest or sample preparation, prior to analysis [1,8,9,10,26], such as ambient temperature, +5 °C, −4 °C, −20 °C, and −29 °C. However, the length of time prior to analysis of the stored samples is seldomly reported. Some studies did not report the storage temperature of natural ergot contaminated samples prior to or after analysis [27,28,29,30].

In an agricultural setting, storage factors for crops consist of temperature, moisture, pests including fungi [31], and time [10]. Stored crops with ergot contamination may have an economic impact on producers through crop quality [2] or livestock consumption [32]. Harvested grain may be downgraded to animal feed if it is contaminated with ergot [12], and livestock may show a reduction in milk yield and weight if ergot contaminated feed is consumed [33]. The effects of storage factors on the concentration of ergot alkaloids could have practical implications if the stored crop quality is impacted.

Assessing the effects of various storage temperatures over an extended time on ergot alkaloid concentrations is limited. Studies with extended storage periods only assessed one temperature, and a study assessing multiple temperatures did not assess the effects of an extended time period at those temperatures. Furthermore, the quantification and investigation of the effect of long-term storage and temperature on multiple *R* and *S* epimers separately has not been assessed to the authors knowledge. It is important to quantify and assess the *S*-epimers of ergot alkaloids because of the recent recommended guideline changes to include the *S*-epimers and the potential bioactivity of the *S*-epimers. Understanding the *R* and *S*-epimer concentration stability in natural contaminated grain over time at multiple temperatures may help ascertain ideal storage conditions and determine the storage factors affecting concentration stability. The objective of this research is to assess the effects of storage time and temperature on the six *R* and six *S*-epimers of the common ergot alkaloids in natural ergot-contaminated wheat.

## 2. Results

### 2.1. Total Ergot Concentration

A small pilot study using the ground ergot contaminated samples was conducted with three groups, prior to drying, freeze drying, and heat drying. The percent change after the drying treatments were minimal: 5% and 7% for the freeze and heat drying, respectively. The concentrations of ergot epimers were assessed in all three groups and the drying treatment did not have an affect on the ergot epimer concentrations. Therefore, moisture content would not impact the results of the present study.

**Figure 1:**
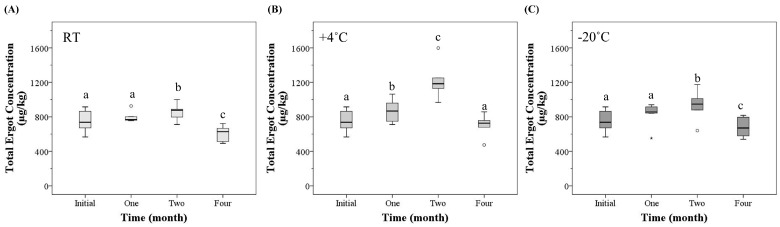
The total (six *R* and six *S*-epimers) ergot concentration (µg/kg) in natural ergot-contaminated hard red spring wheat over time (month) at (**A**) room temperature (RT), (**B**) +4 °C, and (**C**) −20 °C, analyzed utilizing high-performance liquid chromatography–tandem mass spectrometry. [Box-plot: whiskers are defined at the minimum and maximum values, top of box is defined as the 75th percentile, bottom of box is 25th percentile, and middle line is defined at the median. The ° are defined as outliers (>1.5 × interquartile range) and the * are defined as an extreme outlier (>3 × interquartile range)]. All outliers were included in the statistical analysis. Different lowercase letters represent statistical differences between each time period at each temperature (*p* < 0.05, generalized estimating equation, pairwise comparison with sequential Sidak correction, *n* = 6/temperature and time.

Time and temperature influenced the total ergot concentration. There was a significant interaction between the effect of time and temperature on the mean total ergot concentration (MTEC) (*p* < 0.05). At room temperature, +4 °C, and −20 °C storage there was a significant effect of time on the MTEC (*p* < 0.05) (Figure 1). At room temperature, the MTEC did not significantly change after one month of storage, but significantly increased by 15% after two months of storage (*p* = 0.037) and decreased by 19% after four months of storage (*p* < 0.001), compared to the initial analysis. At +4 °C, the MTEC significantly increased over time at one and two months (*p* < 0.001) but returned to the initial concentration after four months. At −20 °C there was a 25% increase at month two (*p* < 0.001) and a 9% decrease at month four (*p* = 0.006), compared to the initial analysis. The specific ergot alkaloids that are influencing the total ergot concentration can be found below.

### 2.2. Total R and S-epimer Concentration

**Figure 2:**
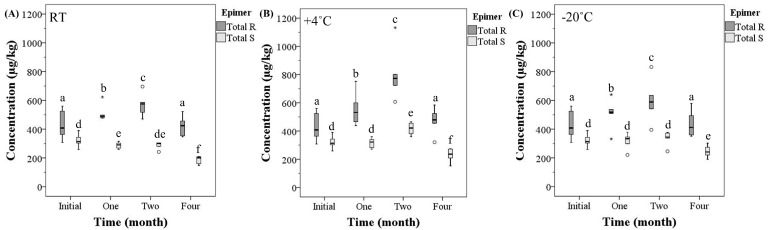
The concentration (µg/kg) of the total *R*-epimers and total *S*-epimers in natural ergot-contaminated hard red spring wheat over time (month) at (**A**) room temperature (RT), (**B**) +4 °C, and (**C**) −20 °C, analyzed utilizing high-performance liquid chromatography–tandem mass spectrometry. [Box-plot: whiskers are defined at the minimum and maximum values, top of box is defined as the 75th percentile, bottom of box is 25th percentile, and middle line is defined at the median. The ° are defined as outliers (>1.5 × interquartile range) and the * are defined as an extreme outlier (>3 × interquartile range)]. All outliers were included in the statistical analysis. Different lowercase letters represent statistical differences between each time period at each temperature for either the total *R*-epimers (a–c) or total *S*-epimers (d–f) (*p* < 0.05, generalized estimating equation, pairwise comparison with sequential Sidak correction, *n* = 6/temperature and time for each epimer.

There was a time effect on the mean total *R* and mean total *S*-epimer concentrations (MTRC and MTSC, respectively) at each storage temperature (*p* < 0.05). From the initial analysis, the MTRC significantly increased by 19–87% at month one and two at room temperature (*p* = 0.01, *p* < 0.001), +4 °C (*p* < 0.001, *p* < 0.001) and −20 °C (*p* = 0.007, *p* < 0.001). At month four, the MTRC was not significantly different from the initial analysis at each temperature (*p* > 0.05). For the MTSC, at room temperature there was a 10% and 41% decrease in the MTSC from the initial analysis compared to month one (*p* = 0.006) and month four (*p* < 0.001), respectively. At 4 °C, there was a 30% MTSC increase from the initial analysis compared to month two (*p* < 0.001), and a 28% decrease compared to month four (*p* < 0.001). At −20 °C, the only difference from the initial analysis was a 24% decrease at month four (*p* < 0.001) (Figure 2).

### 2.3. Temperature Effects on Total, Total R, and Total S-epimer Concentration

The effect of storage temperature on the concentration of mean total ergot, mean total *R*-epimers, and mean total *S*-epimers at each time period was analyzed (Table 1). The MTEC was not different between temperature groups at month one (*p* < 0.05). At month two, +4 °C and −20 °C had significantly higher MTEC than room temperature (*p* < 0.001 and *p* = 0.035, respectively). The same results occurred at month four. The MTRC was not significantly different between temperature groups at each time period (*p* > 0.05). With the exception of month two, the +4 °C group was significantly higher than the room temperature and −20 °C groups (*p* < 0.001). The MTSC between temperature groups at each time period demonstrated significant differences (*p* < 0.05). At month two and four, the MTSC was significantly higher at the +4 °C and −20 °C temperature groups (*p* < 0.001), compared to the room temperature group.

### 2.4. Individual Ergot Epimer Concentration

**Figure 3:**
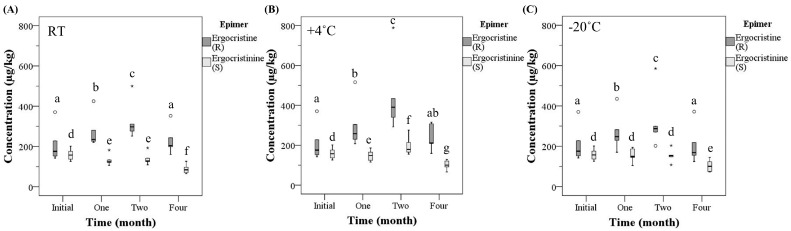
The concentrations (µg/kg) of ergocristine and ergocristinine in natural ergot-contaminated hard red spring wheat over time (month) at (**A**) room temperature (RT), (**B**) +4 °C, and (**C**) −20 °C, analyzed utilizing high-performance liquid chromatography–tandem mass spectrometry. [Box-plot: whiskers are defined at the minimum and maximum values, top of box is defined as the 75th percentile, bottom of box is 25th percentile and middle line is defined at the median. The ° are defined as outliers (>1.5 × interquartile range) and the * are defined as an extreme outlier (>3 × interquartile range)]. All outliers were included in the statistical analysis. Different lowercase letters represent statistical differences between each time period at each temperature for ergocristine (a–c) or ergocristinine (d–g) (*p* < 0.05, generalized estimating equation, pairwise comparison with sequential Sidak correction, *n* = 6/temperature and time for each epimer.

The concentrations of individual epimers analyzed at each temperature group over time were assessed. Ergocristine and ergocristinine had the highest mean concentration compared to the other epimers with an average of 34% and 17%, respectively, of the total ergot concentration (Table S1). Since ergocristine and ergocristinine constituted the greatest percentage of the total ergot concentration, the effects on their concentrations were analyzed. Ergocristine appeared to have influenced the concentration of the total ergot alkaloids to a greater extent, especially at +4 °C in month two, compared to the other ergot alkaloids analyzed. The effects of storage temperature and time on the *R* and *S*-epimers of all other analyzed ergot alkaloids are in the supplemental material (Figures S1–S5). Following the analysis, it was determined that storage time and temperature had an effect on the mean concentrations of ergocristine and ergocristinine (Figure 3). The mean ergocristine concentration increased at month one and two, compared to the initial analysis, independent of temperature (*p* ≤ 0.003), and returned to the initial concentration at month four. The mean ergocristinine concentration decreased at room temperature and +4 °C over time (*p* ≤ 0.005), with the exception at +4 °C in month two. At −20 °C, the concentration did not change from the initial analysis until a decrease at month four (*p* < 0001).

## 3. Discussion

Studies have found time or temperature to have an affect on the concentration of ergot. Specifically, ergot alkaloid concentrations in ergot contaminated matrices have increased [8,9], decreased [10,34], or have shown variation [1] after long term storage. In the present study, the total ergot concentration increased until month two and decreased at month four at all the temperatures evaluated. The total ergot concentration is not stable under certain storage conditions. Previous studies assessing the total ergot concentration have not assessed the effects of time and temperature on the *R* and *S*-epimers separately. One study assessed individual epimer concentrations of harvested and shipped samples; however, the temperature of the stored shipped samples was not provided and not all six common ergot alkaloids were assessed [1]. Similar increases and decreases of the total ergot epimers over time were observed.

The concentration of total ergot alkaloids after storage may vary due to the analysis of not only the *R*-epimers but also the *S*-epimers of ergot alkaloids. An increase in the total *S*-epimer concentration after long term storage has been suggested [8,12], potentially associated with epimerization of the *R*-epimer to the *S*-epimer [1,12,35,36,37,38]. However, the quantification and assessment of the *S*-epimers separately after storage for an extended time at different temperatures was not assessed. The present study demonstrated an increase in the total *R*-epimer concentration until month two, whereas the *S*-epimer concentration mostly decreased or remained stable at all the temperatures. The results observed in the current study do not suggest epimerization of the *R*-epimer to the *S*-epimer. The results may suggest slight back epimerization of the *S*-epimer to the *R*-epimer over time. The *S*-epimer concentrations may be more stable over time compared to the *R*-epimers at various temperatures.

The concentration of specific ergot epimers vary with fungal strain, region, geographical location, year, and host grain type [2,19,39]. Similar to the present study, ergocristine and ergocristinine were found in high concentrations in Canadian grain [1,18,40]. The ergocristine/-inine concentration has previously been reported to be unstable in raw material exposed to different temperatures [13,16]. In the current study, ergocristine concentration varied over time at each temperature, whereas ergocristinine concentration was more stable, especially at −20 °C. Overall, the impact of time and temperature on the ergocristine and ergocristinine concentrations is similar to the pattern observed with the total *R* and total *S*-epimer concentrations. All six common *R* and *S*-epimers of ergot alkaloids should be quantified in ergot contaminated samples since they have different stabilities under the assessed storage conditions.

The rationale for the instability of the concentrations of the total, total *R*, total *S*, and certain individual epimers is unknown. The reason for increase or decrease in the concentrations is out of the scope of the present study. A hypothesis for a decrease in concentrations may be related to the degradation of ergot alkaloids, which has been previously observed [8,35,36,37,38]. The degradation may be associated with the formation of ergot derivatives which can be catalyzed by oxidation, reduction, hydrolysis, and under alkaline or acidic conditions [37]. Microbial activity has also demonstrated ergot alkaloid degradation [8]. In the present study, samples were concealed in airtight tubes, which could produce an anaerobic environment and result in degradation. Other factors such as bacteria or other fungi in the samples may also contribute to the degradation of the ergot alkaloids, which have been noted previously [4]. The samples in the present study were covered in plastic, therefore light degradation should not have been a reason for degradation. The rationale for the increase in concentration is less known. In the studies that also observed an increase in total ergot concentration, this was attributed it to a decrease in ergovaline, in which the ergovaline degradation products would be included in the enzyme-linked immunosorbent assay (ELISA) method for quantification [8,9]. In the present study, an ELISA was not used, therefore, the same rationale cannot be applied. Another study observed a significant increase in total ergovaline after 28 days at −20 °C for particular samples, however, no rationale or discussion was provided [7]. A hypothesis for an increase in concentration in the present study could be that the environmental conditions of the samples, as mentioned above, would increase the extraction efficiency of the specific epimer. However, this hypothesis is purely speculative and would need further assessment and data to support it. The specific configurations of the *R* and *S*-epimers may be more susceptible to degradation, epimerization, or extraction. The variability in the concentrations is likely due to multiple factors.

Ergot contaminated samples are sent routinely to laboratories to determine the concentration of ergot alkaloids [25]. The EU Commission Recommendation on sampling and analysis [41], which has been used to evaluate ergot-contaminated samples [5], reports that storage temperature should not alter the contaminant composition, as such, it does not recommend a specific temperature. A laboratory storage temperature of −20 °C has been suggested so that the concentration of ergot alkaloids does not decrease [7], and this has been implemented [3,42]. However, the suggested storage temperature was only examined for a one-month period. The present study supports the concentration stability of total ergot at −20 °C for one month. However, the total ergot concentration may change in the following months. Since ergot concentrations are not stable under certain storage conditions, time and temperature should be considered when storing natural ergot-contaminated samples in laboratories. Under field conditions, certain storage conditions, such as −20 °C, may not be recommended.

Management and environmental factors during storage in a field setting affect the presence and production of mycotoxins [31]. Ergot contamination of cereal crops and feed intended for selling or livestock consumption is an issue [2,3]. Ergot contaminated matrices may be retained for months before they are utilized [2]. The present study suggests that ergot alkaloids are not stable over time at various temperatures. Storage time and temperature should be considered when storing potential ergot contaminated crops. Changes in the concentration of ergot alkaloids due to storage conditions may affect the quality of the grain or feed. Ergot contaminated samples should be analyzed prior to use to ensure the most reliable estimate of risk assessment.

## 4. Conclusions

Storage time and temperature affect the concentration of total ergot concentration in natural contaminated grain. Total *R* and *S*-epimer concentrations vary at specific temperatures and time periods, and the concentration changes differ from one another. Ergocristine and ergocristinine may influence the change in total *R* and total *S*-epimer concentrations when exposed to the various time and temperatures. The current study does not explicitly demonstrate ideal storage conditions for ergot contaminated samples intended for laboratory or agriculture practices. The rationale for the instability of the ergot alkaloid concentrations is most likely due to multiple abiotic and biotic factors. Long-term storage and various temperatures affect the concentration of ergot alkaloids; therefore, stored grain should be analyzed prior to use to ensure the best ergot alkaloid concentration estimates. Both the *R* and *S*-epimers of the six common ergot alkaloids should be quantified based on varying concentration changes after storage. Storage time and temperature, associated with ergot alkaloid contamination, should be considered for samples intended for consumption.

## 5. Materials and Methods

### 5.1. Sample Preparation

Hard red spring wheat contaminated with ergot (*n* = 6) was obtained from the Canadian Feed Research Centre (North Battleford, SK, Canada). The hard red spring wheat were highly contaminated with ergot sclerotia and were therefore processed as described in detail previously [43]. In summary, the ergot contaminated wheat was diluted with clean wheat to obtain similar starting concentrations among the samples and to achieve concentrations that are observed under practical agricultural situations. The prepared ergot contaminated samples were ground (UDY Cyclone Sample Mill, Model #3010-060, 1 mm mesh, Fort Collins, CO, USA) and hand mixed to ensure a homogenized sample. To ensure a homogenous mixture, six replicates from each of the six samples were analyzed for the concentration of ergot alkaloids. The standard deviation of the six replicates from each of the six prepared samples was relatively low [43]. The starting mean concentration of total ergot alkaloids from the six prepared samples was 841 ± 92 µg/kg. From each prepared ergot contaminated sample, 5 g were weighed(Sartorius BP2100 balance, Elk Grove, CA, USA) and placed directly within a 50 mL plastic trace metal free centrifuge tube and capped.

### 5.2. Pilot Study

A small pilot study was conducted using the samples to assess both the moisture content of the samples and the ergot alkaloid concentrations following freeze and heat drying. This pilot study was used to determine if moisture would have an effect on the results of the present study. Two hard red spring wheat samples, with two replicates each, were weighed (Mettler Toledo NewClassic ML204 balance, Mississauga, ON, Canada) into 50 mL centrifuge tubes and capped. The weight for each sample and replicate was approximately 5 g. This was repeated three times for the samples to be assessed, prior to drying, after freeze drying, and after heat drying. For freeze drying, the centrifuge tubes containing the samples had the caps replaced with Kimwipes and were secured with elastic bands. The samples were placed into a freeze dryer (VirTis Genesis, SP Industries, Warminster, PA, USA) at −20 °C, under vacuum (10–20 m Torr), for 24 h. For heat drying, samples within the centrifuge tubes with the caps removed were placed into adrying oven (Isotemp, Fisher Scientific, Waltham, MA, USA) at +80 °C for 24 h. All samples, including the samples not exposed to drying, were re-weighed into centrifuge tubes and capped for the total ergot alkaloid concentration analysis, which is described below.

### 5.3. Quantification

Concentrations of ergot alkaloids, ergocornine, ergocristine, ergocryptine, ergometrine, ergosine, and ergotamine (*R*-epimers), along with the corresponding -inine epimers (*S*-epimers) (Romer Labs, Tulln, Lower Austria, Austria), were measured in all the prepared ergot contaminated samples using ultra-high-performance liquid chromatography–tandem mass spectrometry (UHPLC-MS/MS) (ThermoFisher Scientific, Waltham, MA, USA) as described previously [43]. Briefly, samples were extracted with acetonitrile:water (80:20) and spun (Benchmixer Multi-tube vortex, Sayreville, NJ, USA). The supernatant was filtered and placed into amber vials along with the internal standard, deuterated lysergic acid diethylamide (Sigma Aldrich, Oakville, ON, Canada). Once the samples were dried down using nitrogen gas (Multivap nitrogen evaporator, Organomation, Berlin, MA, USA ) and reconstituted in methanol:water (50:50), they were ready for analysis. A C18 column was used to separate the epimers. A triple quadrupole with electrospray ionization in positive mode and selective reaction monitoring were used for the mass spectrometry analysis. The method of analysis was validated and described in detail in Cherewyk et al., 2022 [43]. The inter-day precision of the method was <24% relative standard deviation for all quantified analytes.

### 5.4. Experimental Design and Statistical Analysis

An initial quantification (time zero) of the prepared ergot contaminated samples (*n* = 6), with two replicates to assess sampling variability, was conducted to serve as the reference control. The initial reference control sample concentrations were within the standard deviation of the starting mean concentration of the prepared samples. Sub-samples, from each of the prepared ergot contaminated samples, with two replicates were placed at stable room temperature (+22 °C), +4 °C, and −20 °C on the day of the initial quantification. All sub samples used in this study were taken from the starting homogenous prepared ergot contaminated samples. At one, two, and four months after the initial analysis, sub-samples were removed from their respective temperature groups. The samples were analyzed for the concentration of the six *R* and six *S*-epimers as described above. The measured values for the two replicates of each sub sample were averaged. There were six independent samples repeatedly measured for each temperature and time group (*n* = 6/temperature/time). All prepared ergot contaminated samples were covered with black plastic bags to minimize exposure to light.

All statistics were conducted utilizing SPSS 23 (IBM SPSS Statistics for Windows, version 23, IBM Corp., Armonk, NY, USA). A statistical analysis was undertaken using generalized estimating equations (GEEs) with an identity link function and an unstructured correlation matrix. The GEE was used to account for the repeated measures of samples over time and multiple temperatures. The analysis was conducted on the mean total ergot concentration, mean total *R*-epimer concentration, mean total *S*-epimer concentration, and the mean concentration of each individual epimer. Total ergot concentration is defined as all quantified epimers. Total *R*-epimer concentration and total *S*-epimer concentration are the sum of the concentration of the six *R* and six *S*-epimers, respectively. Differences were considered significant at *p* < 0.05. In the presence of a significant interaction between the effects of time and temperature on the mean concentration, GEE was preformed to analyze the effect of time at each temperature. Differences between temperature groups at each time period were also assessed. Multiple pairwise comparisons with a sequential Sidak correction were used to assess differences.

## Figures and Tables

**Table 1:** Effects of temperature at separate time periods on the mean concentration of total ergot, total *R*-epimers, and total *S*-epimers in natural ergot-contaminated wheat analyzed with high-performance liquid chromatography tandem mass spectrometry ^1^.

Time (month)	Temperature (°C)	Mean Total Ergot Alkaloid Concentration	Mean Total *R*-Epimer Concentration	Mean Total *S*-Epimer Concentration
Initial	Initial	749 ± 131	429 ± 99	320 ± 44
1	Room	797 ± 65 ^a^	509 ± 56 ^a^	288 ± 21 ^a^
	+4	870 ± 132 ^a^	554 ± 114 ^a^	316 ± 34 ^b^
	−20	827 ± 140 ^a^	509± 100 ^a^	318 ± 54 ^ab^
2	Room	859 ± 97 ^a^	570 ± 76 ^a^	289 ± 25 ^a^
	+4	1219 ± 210 ^b^	802 ± 177 ^b^	417 ± 41 ^b^
	−20	934 ± 175 ^c^	597 ± 142 ^a^	337 ± 48 ^c^
4	Room	609 ± 89 ^a^	421 ± 66 ^a^	188 ± 27 ^a^
	+4	704 ± 127 ^b^	474 ± 88 ^a^	230 ± 46 ^b^
	−20	679 ± 113 ^b^	435 ± 88 ^a^	245 ± 39 ^c^

^1^ Concentration (µg/kg) values are mean ± standard deviation (*n* = 6). Different letters represent significant differences between temperature groups at each time period. (*p* < 0.05, GEE, Pairwise Comparison, Sequential Sidak correction, *n* = 6).

## Data Availability

The data presented in this study are available through the corresponding author.

## Supplementary Materials

The following supporting information can be downloaded at: https://www.mdpi.com/article/10.3390/toxins15080497/s1. The supplemental materials file includes Figures S1–S5, the effects of time and temperature on the concentration of the other analyzed individual *R* and *S*-epimers; and Table S1, the percentage of each individual epimer compared to the total epimer concentration for each time and temperature group.
