# Supplementary material for: The Impact of Storage Temperature and Time on Ergot Alkaloid Concentrations
Source: Toxins (Basel). 2023 Aug 5;15(8):497. doi: 10.3390/toxins15080497 (PMC10467117; doi:10.3390/toxins15080497)
Supplement: Supplementary file 1 [file toxins-15-00497-s001.zip › toxins-2492690-supplementary.pdf]

# Supplemental Material

# The Impact of Storage Temperature and Time on Ergot Alkaloid Concentrations

**Jensen E. Cherewyk [1,*], Taylor J. Grusie-Ogilvie [2], Sarah E. Parker [3], Barry R. Blakley [1] and Ahmad N. Al-Dissi [4]**

1 Department of Veterinary Biomedical Sciences, Western College of Veterinary Medicine, University of Saskatchewan, Saskatoon, SK S7N 5B4, Canada; brb237@mail.usask.ca
2 Prairie Diagnostic Services (PDS), Saskatoon, SK S7N 5B4, Canada; taylor.ogilvie@pds.usask.ca
3 Centre for Applied Epidemiology, Large Animal Clinical Sciences, Western College of Veterinary Medicine, University of Saskatchewan, Saskatoon, SK S7N 5B4, Canada; sarah.parker@usask.ca
4 Department of Veterinary Pathology, Western College of Veterinary Medicine, University of Saskatchewan, Saskatoon, SK S7N 5B4, Canada; ahmad.aldissi@usask.ca
* Correspondence: jensen.cherewyk@usask.ca

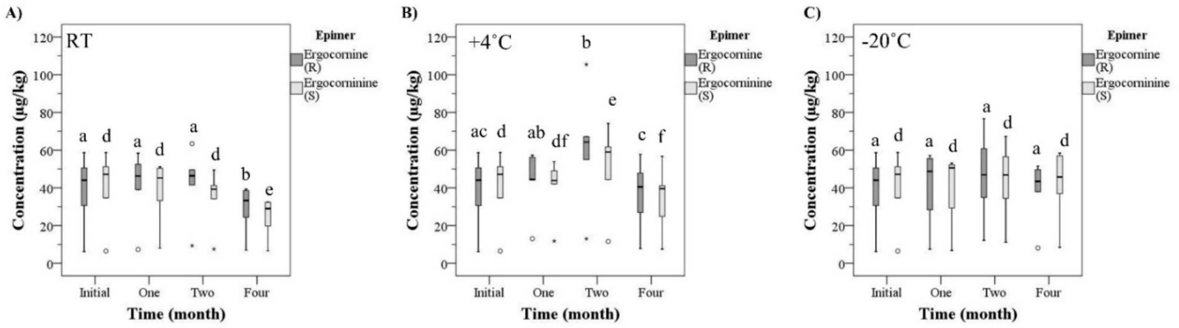


**Figure S1:** The concentration (µg/kg) of ergocornine and ergocorninine in natural ergot-contaminated hard red spring wheat over time (month) at **A)** room temperature (RT), **B)** +4°C, **C)** -20°C, analyzed utilizing high-performance liquid chromatography tandem mass spectrometry. [Box-plot: whiskers are defined at the minimum and maximum values, top of box is defined as the 75th percentile, bottom of box is 25th percentile and middle line is defined at the median. The º are defined as outliers (> 1.5 × interquartile range) and the * are defined as an extreme outlier (> 3 × interquartile range)]. All outliers were included in the statistical analysis. Different lowercase letters represent statistical differences between each time period at each temperature for ergocornine (a-c) or ergocorninine (d-f) ($P < 0.05$, generalized estimating equation, pairwise comparison with sequential Sidak correction, n = 6/temperature and time for each epimer).

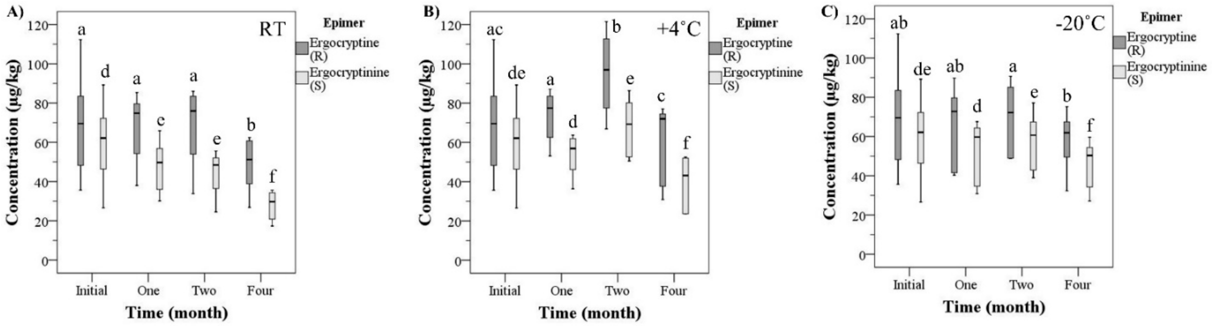


**Figure S2:** The concentration (µg/kg) of ergocryptine and ergocryptinine in natural ergot-contaminated hard red spring wheat over time (month) at **A)** room temperature (RT), **B)** +4˚C, **C)** -20˚C, analyzed utilizing high-performance liquid chromatography tandem mass spectrometry. [Box-plot: whiskers are defined at the minimum and maximum values, top of box is defined as the 75th percentile, bottom of box is 25th percentile and middle line is defined at the median]. Different lowercase letters represent statistical differences between each time period at each temperature for ergocryptine (a-c) or ergocryptinine (d-f) (P < 0.05, generalized estimating equation, pairwise comparison with sequential Sidak correction, n = 6/temperature and time for each epimer).

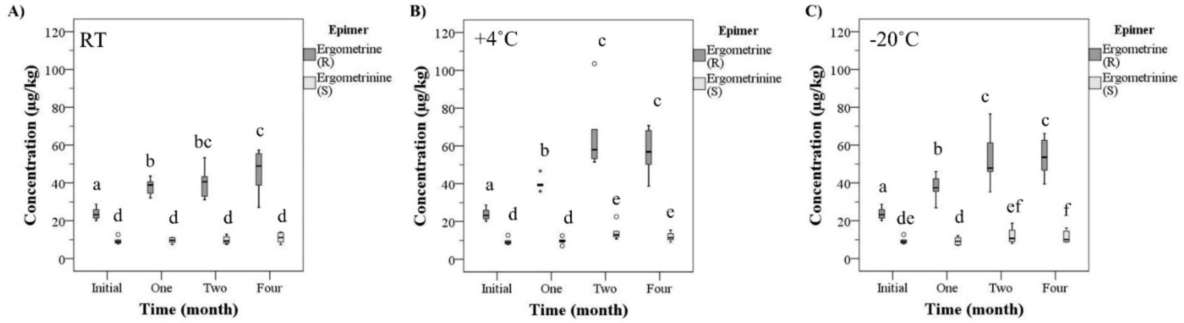


**Figure S3:** The concentration (µg/kg) of ergometrine and ergometrinine in natural ergot-contaminated hard red spring wheat over time (month) at **A)** room temperature (RT), **B)** +4˚C, **C)** -20˚C, analyzed utilizing high-performance liquid chromatography tandem mass spectrometry. [Box-plot: whiskers are defined at the minimum and maximum values, top of box is defined as the 75th percentile, bottom of box is 25th percentile and middle line is defined at the median. The º are defined as outliers (> 1.5 × interquartile range) and the * are defined as an extreme outlier (> 3 × interquartile range)]. All outliers were included in the statistical analysis. Different lowercase letters represent statistical differences between each time period at each temperature for ergometrine (a-c) or ergometrinine (d-f) (P < 0.05, generalized estimating equation, pairwise comparison with sequential Sidak correction, n = 6/temperature and time for each epimer).

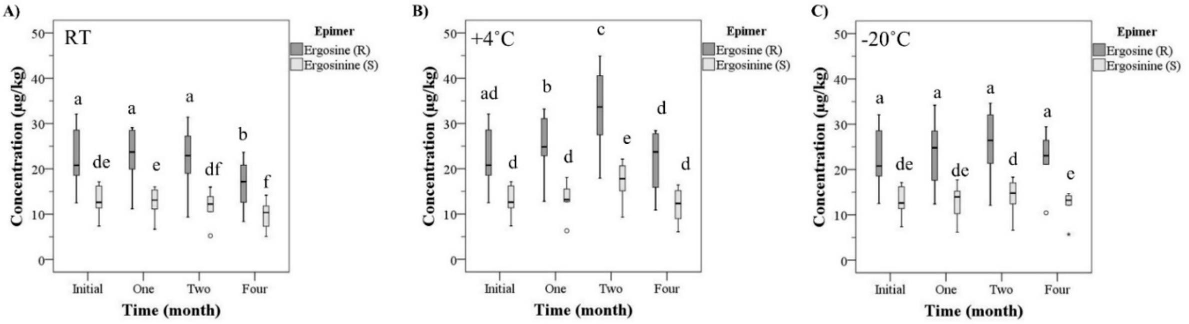


**Figure S4:** The concentration (µg/kg) of ergosine and ergosinine in natural ergot-contaminated hard red spring wheat over time (month) at **A)** room temperature (RT), **B)** +4˚C, **C)** -20˚C, analyzed utilizing high-performance liquid chromatography tandem mass spectrometry. [Box-plot: whiskers are defined at the minimum and maximum values, top of box is defined as the 75th percentile, bottom of box is 25th percentile and middle line is defined at the median. The º are defined as outliers (> 1.5 × interquartile range) and the * are defined as an extreme outlier (> 3 × interquartile range)]. All outliers were included in the statistical analysis. Different lowercase letters represent statistical differences between each time period at each temperature for ergosine (a-d) or ergosinine (d-f) ($P < 0.05$, generalized estimating equation, pairwise comparison with sequential Sidak correction, n = 6/temperature and time for each epimer).

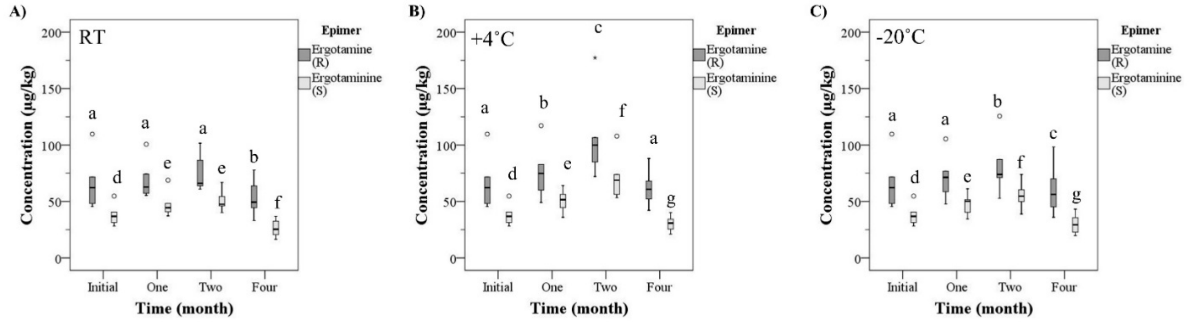


**Figure S5:** The concentration (µg/kg) of ergotamine and ergotaminine in natural ergot-contaminated hard red spring wheat over time (month) at A) room temperature (RT), B) +4˚C, C) -20˚C, analyzed utilizing high-performance liquid chromatography tandem mass spectrometry. [Box-plot: whiskers are defined at the minimum and maximum values, top of box is defined as the 75th percentile, bottom of box is 25th percentile and middle line is defined at the median. The º are defined as outliers (> 1.5 × interquartile range) and the * are defined as an extreme outlier (> 3 × interquartile range)]. All outliers were included in the statistical analysis. Different lowercase letters represent statistical differences between each time period at each temperature for ergotamine (a-c) or ergotaminine (d-g) ($P < 0.05$, generalized estimating equation, pairwise comparison with sequential Sidak correction, n = 6/temperature and time for each epimer).

**Table S1.** Percent (%) of each individual ergot epimer of the total ergot alkaloid concentration.

| Time (month) | Temp (°C) | Ergo cornine | Ergo corninine | Ergo cristine | Ergo cristinine | Ergo cryptine | Ergo cryptinine | Ergo metrine | Ergo metrinine | Ergo sine | Ergo sinine | Ergo tamine | Ergot aminine |
|---|---|---|---|---|---|---|---|---|---|---|---|---|---|
| Initial | Initial | 5.2 | 5.5 | 27.7 | 21.3 | 9.3 | 8.0 | 3.2 | 1.2 | 3.0 | 1.7 | 8.9 | 5.1 |
| 1 | 4 | 5.0 | 4.7 | 34.0 | 17.0 | 8.5 | 6.2 | 4.6 | 1.1 | 2.9 | 1.5 | 8.8 | 5.8 |
| | 20 | 5.0 | 4.9 | 32.5 | 18.7 | 8.0 | 6.4 | 4.5 | 1.1 | 2.9 | 1.6 | 8.7 | 5.8 |
| | 23 | 5.2 | 4.9 | 33.9 | 16.5 | 8.5 | 6.0 | 4.8 | 1.2 | 2.8 | 1.6 | 8.6 | 5.9 |
| 2 | 4 | 5.0 | 4.2 | 36.0 | 16.0 | 7.8 | 5.6 | 5.4 | 1.2 | 2.7 | 1.4 | 8.8 | 5.9 |
| | 20 | 5.0 | 4.7 | 34.5 | 16.5 | 7.5 | 6.2 | 5.6 | 1.3 | 2.7 | 1.5 | 8.6 | 5.9 |
| | 23 | 5.0 | 4.1 | 37.6 | 16.0 | 7.9 | 5.2 | 4.7 | 1.1 | 2.6 | 1.4 | 8.6 | 5.9 |
| 4 | 4 | 5.2 | 5.0 | 33.5 | 14.4 | 8.6 | 5.6 | 8.1 | 1.7 | 3.1 | 1.7 | 8.8 | 4.3 |
| | 20 | 5.7 | 6.2 | 29.6 | 15.2 | 8.5 | 6.8 | 7.9 | 1.7 | 3.3 | 1.8 | 8.9 | 4.4 |
| | 23 | 4.8 | 4.1 | 37.4 | 14.4 | 8.0 | 4.6 | 7.6 | 1.8 | 2.7 | 1.6 | 8.7 | 4.3 |
| Average: | | 5.1 | 4.8 | 33.7 | 16.6 | 8.3 | 6.1 | 5.6 | 1.3 | 2.9 | 1.6 | 8.7 | 5.3 |
